# Evaluation of *VDR* gene polymorphisms in *Trypanosoma cruzi* infection and chronic Chagasic cardiomyopathy

**DOI:** 10.1038/srep31263

**Published:** 2016-08-09

**Authors:** Daniel A Leon Rodriguez, F David Carmona, Clara Isabel González, Javier Martin

**Affiliations:** 1 Instituto de Parasitología y Biomedicina López-Neyra, IPBLN-CSIC, P.T.S, Granada, Spain; 2 Grupo de Inmunología y Epidemiología Molecular, GIEM, Facultad de Salud, Universidad Industrial de Santander, Bucaramanga, Colombia

## Abstract

Vitamin D is an important modulator of the immune response. It acts over several immune cell types where the Vitamin D receptor (VDR) is expressed. Due to the high relevance of this signaling pathway, several studies have investigated the possible influence of genes involved in the metabolism of Vitamin D and its receptor in different human diseases. Here, we analyzed whether four single-nucleotide polymorphisms of the *VDR* gene (rs731236, rs7975232, rs1544410 and rs2228570) are involved in the susceptibility to infection by *Trypanosoma cruzi* and/or to chronic Chagas cardiomyopathy (CCC) in a Colombian endemic population for this parasite. Our results showed that the rs2228570*A allele is associated with CCC development (P = 4.46E−03, OR = 1.51). In summary, the data presented in this report suggest that variation within the *VDR* gene may affect the immune response against *T. cruzi*, increasing the probability of cardiac complications in infected individuals.

Vitamin D is an important modulator of the immune system^1^,^2^. This molecule is involved in both the innate and adaptive responses acting on a wide spectrum of immune cells, in which the Vitamin D receptor (VDR) is expressed^3^. VDR is a member of the nuclear receptor superfamily. Binding of this molecule with the activated form of Vitamin D (1,25(OH)_2_D_3_) causes its dimerization with retinoid X receptor (RXR), which control the transcription of specific genes by interacting with the RNA polymerase II^1^,^4^.

Chagas disease represents an infectious condition caused by the flagellated protozoan *Trypanosoma cruzi* that currently affects around 6 million people in Latin America^5^. Although infected people do not usually develop further complications after the acute phase of disease, up to one third will suffer from chronic Chagas cardiomyopathy (CCC) a condition that affects the life quality and could lead to premature death^6^,^7^. The exact causes of the differential disease outcomes are largely unknown, but increasing knowledge suggests that immune gene variants could play a relevant role by influencing the adequate inflammatory response against the parasitic invasion^8^,^9^,^10^,^11^,^12^. Consistent with this, several members of the inflammatory pathway have been recently associated with risk of infection by *T. cruzi* and/or development of CCC, including genes encoding for HLA class-II molecules (DRB1 and DQB1), the chemokine receptor CCR5, interferon-gamma (IFN-γ), tumor necrosis factor alpha (TNF-α), migration inhibitory factor (MIF), and the interleukins IL-1, IL-12B, IL-17A, IL-18, among others^13^,^14^,^15^,^16^,^17^,^18^,^19^,^20^.

Due to the high relevance of Vitamin D and its receptor in the immune homeostasis, several studies have investigated the possible influence of genes involved in their metabolism in the development of autoimmune conditions and infectious diseases^2^,^21^. In this regard, some polymorphisms of the *VDR* gene have been reported to be associated with different outcomes in autoimmune and infectious diseases, especially in tuberculosis^4^,^21^.

Taking the above into consideration, we aimed to explore for the first time the possible implication of *VDR* genetic variation in Chagas disease susceptibility and clinical manifestation.

## Results

**Figure 1:**
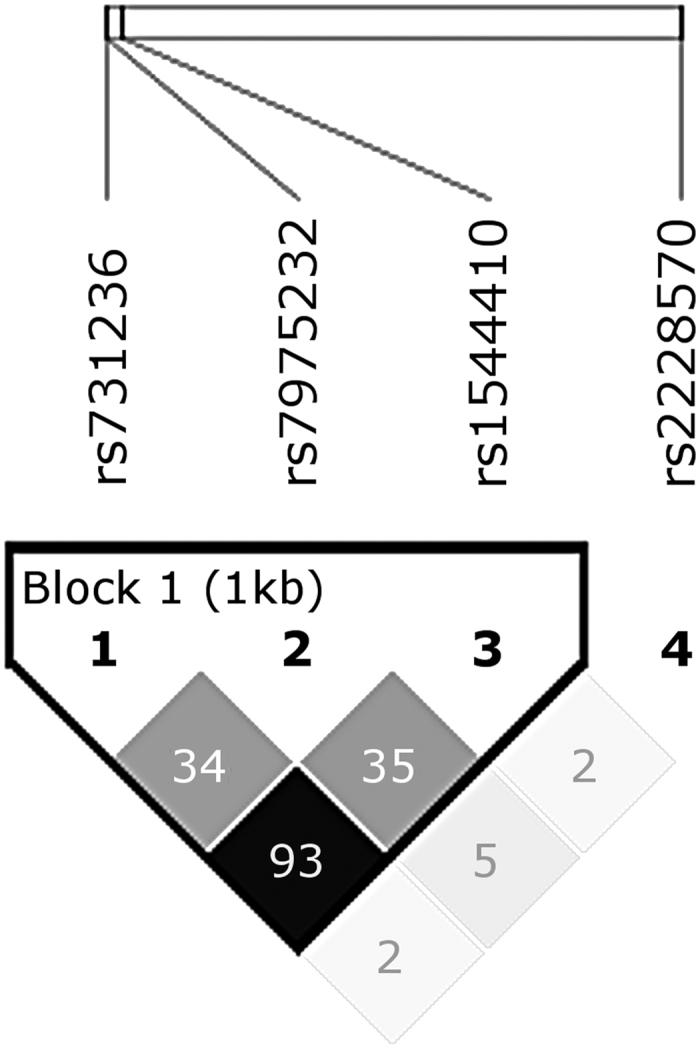
R-Squared plot of analyzed VDR gene variants estimated by using expectation-maximization algorithm in Haploview V4.2.

The genotype frequencies of the four analyzed *VDR* variants did not deviate from Hardy-Weinberg equilibrium in the different subsets (P > 0.01), and their genotyping success rate was over 95%. The statistical power of this study is detailed in Table 1. The SNPs rs731236, rs7975232 and rs1544410 showed a relatively high LD in our study population (Fig. 1). Particularly, rs731236 and rs1544410 had an r^2^ value = 0.93. On the contrary, rs2228570 had an r^2^ value <0.10 with the other SNPs.

First, to analyze the possible implication of the *VDR* polymorphisms in the susceptibility to infection by *T. cruzi*, the allelic and genotypic frequencies of seronegative and seropositive individuals were compared (Table 2). No statistical significance was observed for rs731236, rs7975232 and rs1544410, indicating that these variants may not influence the risk of infection by *T. cruzi* in the studied population. On the other hand, the allele frequencies of rs2228570 differed significantly between the seronegative and seropositive groups (P = 0.0287, OR = 0.81, 95% CI = 0.67−0.98). The minor allele rs2228570*A was overrepresented in the seronegative subset (45.41% vs. 39.94%), suggesting a possible protective effect of this variant against infection by *T. cruzi*. However, the statistical significance was lost after correction for multiple testing (P = 0.1147).

Next, we evaluated the possible association between the *VDR* SNPs and the susceptibility to develop CCC. For that, we compared the allelic and genotypic frequencies of asymptomatic and CCC patients (Table 3). Similar to that observed in the previous analysis, no differences in the allele frequencies of both subgroups of patients were observed for the analyzed SNPs except for rs2228570 (P = 4.46E−03, OR = 1.51, 95% CI = 1.14−2.00). This association was maintained after controlling for multiple testing (P = 0.0178). In this case, the frequency of the rs2228570*A allele was reduced in the asymptomatic patients (34.41% vs. 42.47%), suggesting a putative protective role of this variant against CCC development.

On the other hand, a possible haplotypic effect of the studied *VDR* SNPs was also tested. Due to the high LD in this population (Fig. 1), only three haplotypes were observed in the studied individuals (rs731236|rs7975232|rs1544410: ACC, GAT and AAC). No evidence of association was observed for any haplotype in the different tests performed (seronegative vs. seropositive and asymptomatic vs. CCC, data not shown).

## Discussion

In this study, four genetic variants of the *VDR* gene were tested for association with risk of infection by *T. cruzi* and/or development of CCC in a population from a Colombian endemic region of Chagas disease. Our data provides strong evidence that the *VDR* SNP rs2228570 is associated with CCC in infected individuals, as the odds of having the minor allele was significantly increased in symptomatic individuals as compared to asymptomatic patients. This polymorphism is located at the 5′ end of the *VDR* coding sequence. It has been reported that the presence of the minor allele originates an alternative starting transcription site, which leads to a longer isoform with a reduced transcription activity. Therefore, it has been proposed that the rs2228570 variant may affect the responsiveness to vitamin D^22^,^23^.

Vitamin D is a key molecule of the immune system. The active form of this compound seems to potentiate phagocytosis by macrophages and the production of antimicrobial peptides^1^,^2^. Additionally, it has been observed that it shifts the immune response from TH1/TH17 towards TH2 by inhibiting the production of IFN-γ, IL-12, IL-17, and IL-21, among other cytokines^2^. In Chagas disease, the infection by *T. cruzi* causes a coordinated immune response. That is, in the first line of defense, the innate response is initiated by dendritic cells and macrophages, which produce IL-12 and TNF-α after recognizing the parasite. These cytokines activate natural killer cells that, in turn, enhance the production on IFN-γ. As a consequence, the parasite clearance is facilitated by shifting to a TH1 dominant response that controls the infection^8^. Hence, proinflammatory cytokines, such as IFN-γ, IL-12, IL-17 and TNF-α, are essential for controlling the parasite^24^,^25^,^26^,^27^,^28^. In addition, several studies have suggested that the persistence of *T. cruzi* in the organism is directly related to the development of severe complications in Chagas disease^29^,^30^,^31^. Our results are in agreement with the hypothesis that a stronger immune response may protect patients against the persistence of the parasite. Our data showed that the rs2228570*A allele, which allows the transcription of the enlarged VDR isoform, is more prevalent in CCC patients compared with asymptomatic individuals. We speculate that vitamin D could influence the inflammatory response against *T. cruzi* by downregulating the expression of pro-inflammatory cytokines such as IFN-γ, IL-12 and IL-17. As a consequence, the parasitic persistence could be favored thus increasing the predisposition to develop cardiac complications in Chagas patients. The analysis of vitamin D profiles in individuals exposed to *T. cruzi* infection with different degrees of cardiac involvement would shed light into this idea.

On the other hand, the frequency of the VDR allele rs2228570*A was increased in the seronegative group compared to the seropositive one. In principle, this may be contradictory to the above, as a more potent innate response should lead to a quicker clearance of the parasite in infected individuals before the production of antibodies. However, the statistical significance of this result was lost after correction for multiple testing, which indicates a lack of consistency of this putative association. Further analyses in larger populations are required to clarify this issue.

The role of vitamin D in other protozoan infections is not clear. In malaria, for instance, two different studies indicated that this molecule plays an important role in the control of the immune pathogenesis and cerebral malaria^32^,^33^,^34^. Vitamin D was observed to reduce the risk of cerebral malaria in mice, thus suggesting that it may attenuate the inflammatory response leading to an increase of survival rates^32^. However, evidences also point to vitamin D deficiency as directly responsible for severe cerebral malaria in children coming from Uganda^33^. To our knowledge, only one study has evaluated the possible association between *VDR* genetic variation and malaria. Specifically, two *VDR* SNPs, rs731236 and rs1544410, were shown to affect gametocytemia levels in individuals infected with *Plasmodium vivax*^34^. In Leishmania, two different studies indicated that suppression of both vitamin D and VDR in mice favor the parasite eradication in a TH1 dependent manner^35^,^36^, supporting our hypothesis that vitamin D likely promotes parasite clearance in Chagas.

Regarding the study of *VDR* gene polymorphisms in other infectious diseases, rs2228570 has been associated with tuberculosis in populations of different ethnicities, although the results are contradictory. For instance, a meta-analysis and a case/control study showed that the rs2228570*A allele conferred risk to this condition in Chinese and Iranian patients, respectively^37^,^38^. However, other studies reported associations of this same allele with protection against pulmonary tuberculosis in a Moroccan population^39^. *VDR* rs2228570 has been evaluated also in leprosy, an infectious disease caused by another species of *Mycobacterium*. In this case, homozygosity of rs2228570*A was associated with a higher risk to develop leprosy in an Indian population^40^.

Thus, it seems clear that the vitamin D signaling has a major role in the immune response against different infectious diseases, and this has led to propose that controlling the uptake and metabolic status of vitamin D may be useful to improve the current therapeutic strategies in these conditions^1^,^41^. Consequently, future studies aimed to analyze the levels of vitamin D in the different groups of individuals included in this study, could represent an important step forward towards the understanding of Chagas disease development and treatment. Replication of the genetic results reported here in larger independent cohorts would be also desirable to confirm our findings.

In conclusion, we have observed a genetic association between the *VDR* gene polymorphism rs2228570 and risk to develop CCC in Chagas patients. Although this association has a clear functional implication, more powered studies and functional experiments are needed to definitively confirm the involvement of the vitamin D signaling in the development of this severe complication after infection by *T. cruzi*.

## Material and Methods

### Study subjects

For this study, 1,172 individuals from the endemic regions for *T. cruzi* Guanentina and Comunera at the Santander Department, Colombia (localized between 5°26′ and 8°08′ north and 72°26′ and 74°32′ west) (Supplementary Figure S1) were enrolled. These provinces are located on the north-east side of the country, and their population is a homogeneous mix with no specific concentration of any ethnicity. Regarding the selection criteria, participants were recruited either after a medical visit to the endemic area or after attending to “Fundación Cardiovascular de Colombia”, a medical institution specialized in cardiovascular disorders situated in the city of Floridablanca (Supplementary Figure S1), where they were diagnosed with Chagas disease. Almost all invited individuals agreed to participate in this study. Since Chagas disease is a chronical disorder in which symptoms may appear after several years of infection^7^,^8^, we decided to exclude those individuals younger than 30 years old (189 in total) in order to perform a more consistent analysis of possible resistance to the infection (as the selected individuals had a longer time of exposure to vectorial infection). In total, 983 individuals were finally included in the analyses. Then, the individuals were classified as seronegative or seropositive (n = 436 and 547, respectively) for *T. cruzi* antigens accordingly to two different commercial immunological tests, the enzyme-linked immunosorbent assay (ELISA) (BioELISA Chagas, Biokit, Lliçà d’Amunt, Barcelona, Spain) and indirect hemagglutination (Chagatest IHA, Wiener Lab, Rosario, Argentina). All seronegative individuals were negative for both tests. Seropositive patients underwent an exhaustive clinical evaluation and were subsequently subdivided into asymptomatic and chronic Chagas cardiomyopathy (CCC) patients (n = 171 and 376, respectively) based on electrocardiogram and echocardiogram information. This classification is based in the guidelines from the World Health Organization (WHO) (http://www.who.int/mediacentre/factsheets/fs340/en/), the Pan-American Health Organization (PAHO) (http://www.paho.org/hq/index.php?option=com_topics&view=article&id=10&Itemid=40743), and the classification established by the international consensus of Buenos Aires of 2010 (http://www.fac.org.ar/7cvc/llave/c016/mordinio.pdf). The mean age of participants was 52.11 years for seronegative individuals, 56.67 for asymptomatic individuals and 62.66 for CCC patients. The sex distribution for the entire group was 55% female and 45% male.

### Ethics statement

All participants signed an informed consent. This study was approved by the Act No. 15 of 2005 by the Ethics Committees from “Universidad Industrial de Santander” and “Fundación Cardiovascular de Colombia” in accordance with the ethical standards laid down in the 1964 Declaration of Helsinki.

### SNP selection

Following a candidate gene strategy, four single-nucleotide polymorphisms (SNPs) of the *VDR* gene, previously described to have a functional implication on gene expression and function, were selected for this study^22^,^23^,^42^,^43^. These variants include rs731236 (Taq1), rs7975232 (Apa1), rs1544410 (Bsm1), and rs2228570 (Fok1). The three first SNPs have been related to a differential expression of VDR in different cells, whereas the latter represents a cytosine to thymine change in an ACG codon, which creates an alternative start site that produces a longer VDR protein with a reduced transcriptional activity^22^,^23^,^42^,^43^.

### DNA extraction and Genotyping

Genomic DNA was isolated from 5–10 ml of EDTA anticoagulated blood samples using standard salting-out techniques as previously described^44^. The four SNPs were genotyped using TaqMan allelic discrimination assays from Applied Biosystems (Foster City, California, USA; catalog numbers: C___2404008_10, C__28977635_10, C___8716062_10, C__12060045_20). The genotyping was performed on a LightCycler 480 real-time PCR system (Roche Diagnostics, Basel, Switzerland).

### Statistical analysis

All the statistical analyses were performed with the statistical software package Plink V1.07 (http://pngu.mgh.harvard.edu/purcell/plink)^45^. For all groups of individuals, possible deviance from Hardy-Weinberg equilibrium was determined in every SNP at the 1% significance level. To test for possible allelic and genotypic associations with disease susceptibility and clinical complications, we compared the allelic, genotypic and haplotypic frequencies of the *VDR* variants between seronegative vs. seropositive individuals and asymptomatic vs. CCC individuals by logistic regression assuming an additive model and using age as covariate (as in Chagas disease symptoms may appear many years after infection). The Benjamini & Hochberg step-up false discovery rate (FDR) correction was used in all analyses to control for possible multiple testing effects. Odds ratios (OR) and 95% confidence intervals (CI) were calculated according to Woolf’s method. P-values lower than 0.05 were considered as statistically significant. Pairwise linkage disequilibrium (LD) (D’ and r2) and haplotypic blocks were estimated using an expectation–maximization algorithm as implemented in Haploview v4.2^46^. The statistical power of our study was calculated with the Power Calculator for Genetic Studies 2006 (CaTS) software (http://www.sph.umich.edu/csg/abecasis/CaTS/)^47^.

## Additional Information

**How to cite this article**: Leon Rodriguez D. A. *et al*. Evaluation of *VDR* gene polymorphisms in *Trypanosoma cruzi* infection and chronic Chagasic cardiomyopathy. *Sci. Rep*. **6**, 31263; doi: 10.1038/srep31263 (2016).

## Supplementary Material

Supplementary Information

## Figures and Tables

**Table 1:** Statistical power calculation of our study considering three different OR.

Statistical power calculation		
	*T. cruzi* infection (436/547)*	Chronic Chagasic cardiomyopathy (171/376)**
OR = 1.50	98%	82%
OR = 1.25	60%	35%
OR = 1.10	16%	10%

The estimation was performed considering a prevalence of 1.44% and a minor allele frequency of 25%.

^*^Analysis performed by using 436 seronegative vs. 547 seropositive individuals.

^**^Analysis performed by using 171 asymptomatic vs. 376 chronic Chagas cardiomyopathy individuals.

**Table 2:** Logistic regression analysis of *VDR* polymorphisms in seronegative and seropositive individuals including age as covariate.

SNP	1|2	Group (N)	Genotype. N (%)			MAF %	Allele test/Age		
			1|1	1|2	2|2		P	P_FDR_	OR [95% CI]
**rs731236**	G|A	Seronegative (435)	42 (9.66)	166 (38.16)	227 (52.18)	28.74			
		Seropositive (545)	43 (7.89)	228 (41.83)	274 (50.28)	28.81	0.9024	0.9024	1.01 [0.83–1.24]
**rs7975232**	C|A	Seronegative (436)	96 (22.02)	233 (53.44)	107 (24.54)	48.74			
		Seropositive (532)	97 (18.23)	291 (54.70)	144 (27.07)	45.58	0.2206	0.4412	0.88 [0.72–1.08]
**rs1544410**	T|C	Seronegative (434)	39 (8.99)	166 (38.25)	229 (52.76)	28.11			
		Seropositive (535)	43 (8.04)	227 (42.43)	265 (49.53)	29.25	0.4677	0.6236	1.08 [0.88–1.33]
**rs2228570**	A|G	Seronegative (436)	89 (20.41)	218 (50.00)	129 (29.59)	45.41			
		Seropositive (542)	83 (15.31)	267 (49.26)	192 (35.42)	39.94	**0.0287**	0.1147	0.81 [0.67–0.98]

**Table 3:** Logistic regression analysis of *VDR* polymorphisms in asymptomatic and chronic Chagas cardiomyopathy (CCC) individuals. including age as covariate.

SNP	1|2	Group (N)	Genotype. N (%)			MAF %	Allele test/Age		
			1|1	1|2	2|2		P	P_FDR_	OR [95% CI]
**rs731236**	G|A	Asymptomatic (170)	17 (10.00)	69 (40.59)	84 (49.41)	30.29			
		CCC (375)	26 (6.93)	159 (42.40)	190 (50.67)	28.13	0.4406	0.4633	0.89 [0.67–1.19]
**rs7975232**	C|A	Asymptomatic (164)	28 (17.07)	88 (53.66)	48 (29.27)	43.90			
		CCC (368)	69 (18.75)	203 (55.16)	96 (26.09)	46.33	0.4304	0.4633	1.12 [0.84–1.49]
**rs1544410**	T|C	Asymptomatic (168)	16 (9.52)	72 (42.86)	80 (47.62)	30.95			
		CCC (367)	27 (7.36)	155 (42.23)	185 (50.41)	28.47	0.4633	0.4633	0.90 [0.67–1.20]
**rs2228570**	A|G	Asymptomatic (170)	21 (12.35)	75 (44.12)	74 (43.53)	34.41			
		CCC (372)	69 (16.67)	192 (51.61)	118 (31.72)	42.47	**4.46E-03**	**0.0178**	1.51 [1.14–2.00]
