# Supplementary material for: Evaluation of VDR gene polymorphisms in Trypanosoma cruzi infection and chronic Chagasic cardiomyopathy
Source: Sci Rep. 2016 Aug 9;6:31263. doi: 10.1038/srep31263 (PMC4977507; doi:10.1038/srep31263)

Supplementary information

Manuscript Title

Evaluation of *VDR* gene polymorphisms in *Trypanosoma cruzi* infection and chronic Chagasic cardiomyopathy

Authors

Daniel A Leon Rodriguez, F David Carmona, Clara Isabel González and Javier Martin

Supplementary Figure S1. Map of Colombia indicating the endemic areas of this study; grey, Santander Department; red, Guanentina and Comunera Provinces; X, city of Floridablanca. This map was downloaded from https://commons.wikimedia.org/wiki/File:Colombia_departments_blank.png and modified with GIMP™ V2.8.16 (https://www.gimp.org/). The map was modified from the image available under the Creative Commons Attribution-ShareAlike License at https://creativecommons.org/licenses/by-sa/3.0/deed.en and originally published by https://commons.wikimedia.org/wiki/User:Golbez.

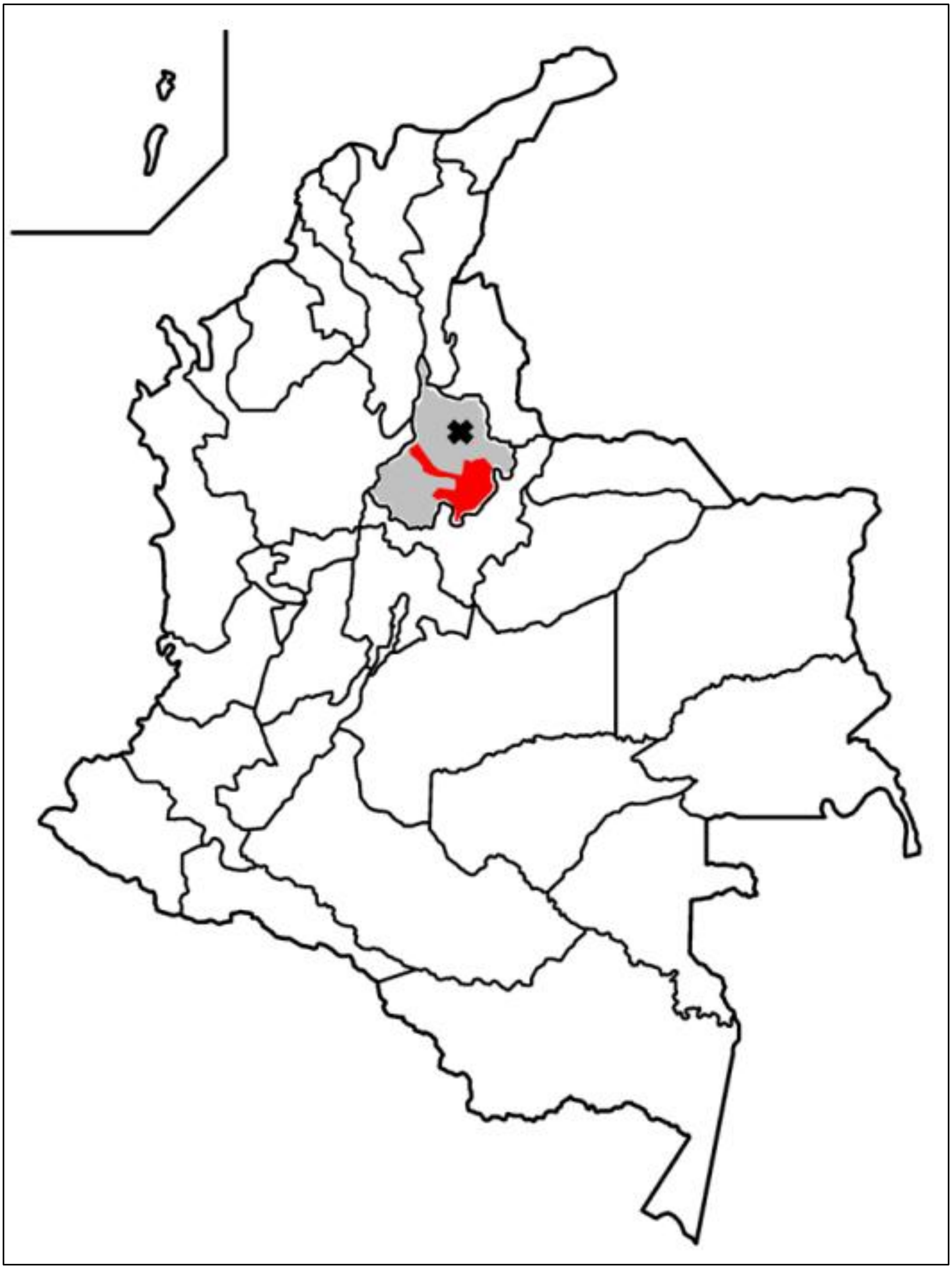

Supplement: Supplementary Information [file srep31263-s1.pdf]
